# Preliminary studies of biosynthesis of heme O and heme A in intraerythrocytic stages of *P. falciparum*

**DOI:** 10.1186/1475-2875-13-S1-P82

**Published:** 2014-09-22

**Authors:** Raquel Simão-Gurge, Julia Cricco, Gerhard Wunderlich, Emilia Kimura, Brenda Cirulli, Alejandro Katzin

**Affiliations:** 1 University of São Paulo, São Paulo, Sp, Brazil; 2 The National University of Rosario, Rosario, Argentina

## Background

Intermediates of the isoprenoid metabolism can bind to heme groups. Heme is critical and its derivatives, heme O and heme A, are the main compounds of aerobic respiration. They are synthesized by farnesylation of heme groups by “heme O synthase” (HOS or Cox10) and “heme A synthase” (HAS or Cox15). The identification of heme O and heme A and characterization of enzymes involved in their synthesis are important for exploring the possible products biosynthesized by the parasite derived from the MEP pathway. Importantly, hemes A and O are essential molecules for numerous living organisms, since they are critical components of the mitochondrial electron transport chain, catalyzing the reduction of O_2_ to H_2_O. Additionally, the heme group is an important target of drugs such as artemisinin and quinolines.

## Methods

The characterization of heme O in *P. falciparum* was done by two different methods using metabolic labeling with [^3^H] FPP and [^14^C] Glycine followed by extraction and separation through a C18 column permitting to observe heme O as well as heme B biosynthesis. The plasmodial orthologs of HOS (PfCox10) and HAS (PfCox15) genes were identified by their homology to HOS and HAS enzymes from other organisms. We then genetically tagged both genes with GFP and HA coding sequences and visualized transfectants by fl uorescence microscopy.

## Results and conclusion

We observed production of heme O and heme B in blood stage parasites. Visualization tagged PfCox10 and PfCox15 suggested that both enzymes were most probably localized in the mitochondria although PfCox10 presented an unusual extension at its N-terminus.

